# Depression and Trauma Symptoms in Children and Adolescents in the Context of the War in Ukraine: The Role of Demographic, Cultural, and Migration-Related Factors

**DOI:** 10.1192/j.eurpsy.2026.10984

**Published:** 2026-07-31

**Authors:** M. Stanisławska-Kubiak, D. Wiśniewska-Szeplewicz, M. Andrusiewicz, H. Katolyk, M. Stanisławska, R. Malak, G. Teusz, E. Mojs, J. Mojs, M. Mojs

**Affiliations:** 1 Department of Clinical Psychology, Poznan University of Medical Sciences; 2 NeuroCare Medical Center; 3 Department of Cell Biology, Poznan University of Medical Sciences; 4 NeuroCare Medical Center, Poznań, Poland; 5 Department of Theoretical Psychology, Institute of Management, Psychology and SafetyPsychology, Lviv State University of Internal Affairs, Lviv, Ukraine; 6 Operational and Reconnaissance Department, Border Guard Academy, Koszalin; 7 Department and Clinic of Rheumatology, Rehabilitation and Internal Medicine, Poznan University of Medical Sciences; 8 Faculty of Educational Studies, Adam Mickiewicz University in Poznan; 9 Department of Clinical Psychology, Poznan University of Medical Sciences; 10 Poznan University of Medical Sciences; 11 Adam Mickiewicz University in Poznan, Poznań, Poland

## Abstract

**Introduction:**

The psychological consequences of war on children are well documented, with previous research highlighting the protective role of caregiver presence in mitigating trauma. However, the ongoing armed conflict in Ukraine provides a new context, requiring analysis of the differential impact of direct war exposure and forced displacement.

**Objectives:**

This study aimed to examine the relationship between depressive and trauma-related symptoms among children and adolescents affected by the war in Ukraine, with a particular focus on demographic, cultural, and migration-related factors.

**Methods:**

A cross-sectional study was conducted with 222 participants aged 7–21 years from Poland (n=81), Ukraine (n=114), and displaced Ukrainian populations (n=23). Depressive symptoms were assessed using the CDI-2, and trauma symptoms were measured with the ITQ-CA. Supplementary data were collected on war exposure, migration experiences, and leisure activities.

**Results:**

Associations between depression and trauma symptoms varied by gender, country of origin, engagement in hobbies, degree of war exposure, and return status to the home country. Direct witnessing of war events was strongly associated with higher trauma symptoms, though not consistently with elevated depression scores. Displaced children exhibited the highest co-occurrence of depressive and trauma symptoms. Engagement in hobbies was linked to lower symptom severity and weaker correlations between depression and trauma indicators.

**Conclusions:**

The findings underscore the need for individualized, culturally sensitive mental health interventions that address both trauma and the secondary consequences of displacement. Strengthening protective factors such as leisure activities may foster resilience among children and adolescents exposed to the consequences of war.

**Disclosure of Interest:**

None Declared

